# Applications of Normal S-Iterative Method to a Nonlinear Integral Equation

**DOI:** 10.1155/2014/943127

**Published:** 2014-08-11

**Authors:** Faik Gürsoy

**Affiliations:** Department of Mathematics, Faculty of Science and Letters, Yildiz Technical University, Davutpasa Campus, Esenler, 34220 Istanbul, Turkey

## Abstract

It has been shown that a normal S-iterative method converges to the solution of a mixed type Volterra-Fredholm functional nonlinear integral equation. Furthermore, a data dependence result for the solution of this integral equation has been proven.

## 1. Introduction

The scientists working in almost every field of science are faced with nonlinear problems, because nature itself is intrinsically nonlinear. Such problems can be modelled as nonlinear mathematical equations. Solving nonlinear equations is, of course, considered to be a matter of the uttermost importance in mathematics and its manifold applications. There are numerous systematic approaches which are classified as direct and iterative methods to solve such equations in the existing literature. Indeed, by using direct methods, finding solutions to a complicated nonlinear equation can be an almost insurmountable challenge. In this context, iterative methods have become very important mathematical tools for finding solutions to a nonlinear equation. For a comprehensive review and references to the extensive literature on the iterative methods, the interested reader may refer to some recent works [1–8].

Recently, Sahu [9] and Khan [10], who was probably unaware of Sahu's work, introduced the following iterative process which has been called normal S-iterative method and Picard-Mann hybrid iterative process by Sahu and Khan, respectively, and hereinafter referred to as the “normal S-iterative method.”

**Definition 1 .**

Let *X* be an ambient space and let *T* be a self-map of *X*. A normal S-iterative method is defined by

(1) x0∈X,xn+1=Tyn,yn=(1−ξn)xn+ξnTxn, n∈N,

where {*ξ*_*n*_}_*n*=0_^*∞*^ is a real sequence in [0,1] satisfying certain control condition(s).

It has been shown both analytically and numerically in [9, 10] that iterative method (1) converges at a rate faster than all Picard [11], Mann [12], and Ishikawa [13] iterative processes in the sense of Berinde [14] for the class of contraction mappings.

This iterative method, due to its simplicity and fastness, has attracted the attention of many researchers and has been examined in various aspects; see [15–20].

In this paper, inspired by the performance and achievements of normal S-iterative method (1), we will give some of its applications. We will show that normal S-iterative method (1) converges strongly to the solution of the following mixed type Volterra-Fredholm functional nonlinear integral equation which was considered in [21]:

(2) x(t)=F(t,x(t),∫a1t1⋯∫amtmK(t,s,x(s))ds,∫a1b1⋯∫ambmH(t,s,x(s))ds),

where [*a*_1_; *b*_1_] × ⋯×[*a*_*m*_; *b*_*m*_] is an interval in *R* ^*m*^, *K*, *H* : [*a*_1_; *b*_1_] × ⋯×[*a*_*m*_; *b*_*m*_] × [*a*_1_; *b*_1_] × ⋯×[*a*_*m*_; *b*_*m*_] × *R* → *R* continuous functions, and *F* : [*a*_1_; *b*_1_] × ⋯×[*a*_*m*_; *b*_*m*_] × *R* ^3^ → *R*.

Also we give a data dependence result for the solution of integral equation (2) with the help of normal S-iterative method (1).

We end this section with some known results which will be useful in proving our main results.

**Theorem 2 (see [21]).**

We suppose that the following conditions are satisfied:

(*A*_1_) *K*, *H* ∈ *C*([*a*_1_; *b*_1_] × ⋯×[*a*_*m*_; *b*_*m*_] × [*a*_1_; *b*_1_] × ⋯×[*a*_*m*_; *b*_*m*_] × *R*);
(*A*_2_) *F* ∈ *C*([*a*_1_; *b*_1_] × ⋯×[*a*_*m*_; *b*_*m*_] × *R* ^3^);
(*A*_3_) there exist nonnegative constants *α*, *β*, and *γ* such that
  (3) |F(t,u1,v1,w1)−F(t,u2,v2,w2)| ≤α|u1−u2|+β|v1−v2|+γ|w1−w2|,

for all *t* ∈ [*a*_1_; *b*_1_] × ⋯×[*a*_*m*_; *b*_*m*_], *u*_*i*_, *v*_*i*_, *w*_*i*_ ∈ *R*, *i* = 1,2;

(*A*_4_) there exist nonnegative constants *L*_*K*_ and *L*_*H*_ such that
  (4) |K(t,s,u)−K(t,s,v)|≤LK|u−v|,|H(t,s,u)−H(t,s,v)|≤LH|u−v|,

for all *t*, *s* ∈ [*a*_1_; *b*_1_] × ⋯×[*a*_*m*_; *b*_*m*_], *u*, *v* ∈ *R*;

(*A*_5_) *α* + (*βL*_*K*_ + *γL*_*H*_)(*b*_1_ − *a*_1_) ⋯ (*b*_*m*_ − *a*_*m*_) < 1.

Then (2) has a unique solution x* ∈ *C*([*a*_1_; *b*_1_] × ⋯×[*a*_*m*_; *b*_*m*_]).

**Lemma 3 (see [22]).**

Let {*β*_*n*_}_*n*=0_^*∞*^ be a nonnegative sequence for which one assumes there exists *n*_0_ ∈ *N*, such that for all *n* ≥ *n*_0_ one has satisfied the inequality

(5) $$\begin{array}{c} \beta_{n+1}\leq \left(1-\mu_{n}\right)\beta_{n}+\mu_{n}\gamma_{n}, \end{array}$$

where *μ*_*n*_ ∈ (0,1), for all *n* ∈ *N*, ∑_*n*=0_^*∞*^ *μ*_*n*_ = *∞*, and *γ*_*n*_ ≥ 0, for all *n* ∈ *N*. Then the following inequality holds:

(6) $$\begin{array}{c} 0\leq \lim\underset{n\to \infty }{\sup}\beta_{n}\leq \lim\underset{n\to \infty }{\sup}\gamma_{n}. \end{array}$$

## 2. Main Results

**Theorem 4 .**

One opines that all conditions (*A*_1_)–(*A*_5_) in Theorem 2 are performed. Let {*ξ*_*n*_}_*n*=0_^*∞*^ ⊂ [0,1] be a real sequence satisfying ∑_*n*=0_^*∞*^ *ξ*_*n*_ = *∞*. Then (2) has a unique solution, say x*, in *C* ([*a*_1_; *b*_1_] × ⋯×[*a*_*m*_; *b*_*m*_]) and normal S-iterative method (1) converges to x*.

**Proof**

We consider the Banach space *B* = *C* ([*a*_1_; *b*_1_] × ⋯×[*a*_*m*_; *b*_*m*_], ||·||_*C*_), where ||·||_*C*_ is Chebyshev's norm. Let {*x*_*n*_}_*n*=0_^*∞*^ be an iterative sequence generated by normal S-iterative method (1) for the operator *A* : *B* → *B* defined by

(7) A(x)(t)=F(t,x(t),∫a1t1⋯∫amtmK(t,s,x(s))ds, ∫a1b1⋯∫ambmH(t,s,x(s))ds).

We will show that *x*_*n*_ → x* as *n* → *∞*.

From (1), (2), and assumptions (*A*_1_)–(*A*_4_), we have that

(8) ||xn+1−x∗||=||Ayn−x∗||=|A(yn)(t)−A(x∗)(t)|=|F(t,yn(t),∫a1t1⋯∫amtmK(t,s,yn(s))ds, ∫a1b1⋯∫ambmH(t,s,yn(s))ds)  −F(t,x∗(t),∫a1t1⋯∫amtmK(t,s,x∗(s))ds, ∫a1b1⋯∫ambmH(t,s,x∗(s))ds)|≤α|yn(t)−x∗(t)| +β|∫a1t1⋯∫amtmK(t,s,yn(s))ds −∫a1t1⋯∫amtmK(t,s,x∗(s))ds| +γ|∫a1b1⋯∫ambmH(t,s,yn(s))ds −∫a1b1⋯∫ambmH(t,s,x∗(s))ds|≤α|yn(t)−x∗(t)| +β∫a1t1⋯∫amtm|K(t,s,yn(s))−K(t,s,x∗(s))|ds +γ∫a1b1⋯∫ambm|H(t,s,yn(s))−H(t,s,x∗(s))|ds≤α|yn(t)−x∗(t)|+β∫a1t1⋯∫amtmLK|yn(s)−x∗(s)|ds +γ∫a1b1⋯∫ambmLH|yn(s)−x∗(s)|ds≤[α+(βLK+γLH)∏i=1m(bi−ai)]||yn−x∗||,

(9) ||yn−x∗||≤(1−ξn)|xn(t)−x∗(t)| +ξn|A(xn)(t)−A(x∗)(t)|=(1−ξn)|xn(t)−x∗(t)| +ξn|F(t,xn(t),∫a1t1⋯∫amtmK(t,s,xn(s))ds, ∫a1b1⋯∫ambmH(t,s,xn(s))ds) −F(t,x∗(t),∫a1t1⋯∫amtmK(t,s,x∗(s))ds, ∫a1b1⋯∫ambmH(t,s,x∗(s))ds)|≤(1−ξn)|xn(t)−x∗(t)|+ξnα|xn(t)−x∗(t)| +ξnβ∫a1t1⋯∫amtmLK|xn(s)−x∗(s)|ds +ξnγ∫a1b1⋯∫ambmLH|xn(s)−x∗(s)|ds≤{1−ξn(1−[α+(βLK+γLH)∏i=1m(bi−ai)])} ×||xn−x∗||.

Combining (8) with (9), we obtain

(10) ||xn+1−x∗||≤[α+(βLK+γLH)∏i=1m(bi−ai)] ×{1−ξn(1−[α+(βLK+γLH)∏i=1m(bi−ai)])} ×||xn−x∗||,

or, from assumption (*A*_5_),

(11) ||xn+1−x∗||≤{1−ξn(1−[α+(βLK+γLH)∏i=1m(bi−ai)])} ×||xn−x∗||.

Thus, by induction, we get

(12) ||xn+1−x||≤||x0−x∗|| ×∏k=0n{1−ξk(1−[α+(βLK+γLH) ×∏i=1m(bi−ai)])}.

Since *ξ*_*k*_ ∈ [0,1] for all *k* ∈ *N*, assumption (*A*_5_) yields

(13) $$\begin{array}{c} 1-\xi_{k}\left(1-\left[\alpha +\left(\beta L_{K}+\gamma L_{H}\right)\overset{m}{\underset{i=1}{\prod }}\left(b_{i}-a_{i}\right)\right]\right)<1. \end{array}$$

Having regard to the fact that *e* ^*x*^ ≥ 1 − *x* for all *x* ∈ [0,1], we can rewrite (12) as

(14) $$\begin{array}{c} ||x_{n+1}-x^{*}||\leq ||x_{0}-x^{*}||e^{-(1-[\alpha +(\beta L_{K}+\gamma L_{H})\prod_{i=1}^{m}(b_{i}-a_{i})])\sum_{k=0}^{n}\xi_{k}}, \end{array}$$

which yields lim⁡_*n*→*∞*_||*x*_*n*_ − x*|| = 0.

We now prove the data dependence of the solution for integral equation (2) with the help of the normal S-iterative method (1).

Let *B* be as in the proof of Theorem 4 and *T*, $\tilde{T}:B\to B$ two operators defined by

(15) T(x)(t)=F(t,x(t),∫a1t1⋯∫amtmK(t,s,x(s))ds, ∫a1b1⋯∫ambmH(t,s,x(s))ds),

(16) T~(x)(t)=F(t,x(t),∫a1t1⋯∫amtmK~(t,s,x(s))ds, ∫a1b1⋯∫ambmH~(t,s,x(s))ds),

where *K*, $\tilde{K}$, *H*, $\tilde{H}\in C\left(\left[a_{1};b_{1}\right]\times \cdots \times \left[a_{m};b_{m}\right]\times \left[a_{1};b_{1}\right]\times \cdots \times \left[a_{m};b_{m}\right]\times R\right)$.

**Theorem 5 .**

Let *F*, *K*, and *H* be defined as in Theorem 2 and let {*x*_*n*_}_*n*=0_^*∞*^ be an iterative sequence defined by normal S-iterative method (1) associated with *T*. Let ${\left\{{\tilde{x}}_{n}\right\}}_{n=0}^{\infty }$ be an iterative sequence generated by

(17) x~0∈B,x~n+1=T~y~n,y~n=(1−ξn)x~n+ξnT~x~n, n∈N,

where *B* is defined as in the proof of Theorem 4 and {*ξ*_*n*_}_*n*=0_^*∞*^ is a real sequence in [0,1] satisfying (i) 1/2 ≤ *ξ*_*n*_, for all *n* ∈ *N*, and (ii) ∑_*n*=0_^*∞*^ *ξ*_*n*_ = *∞*. One supposes further that (iii) there exist nonnegative constants *ε*_1_ and *ε*_2_ such that $\left|K\left(t,s,u\right)-\tilde{K}\left(t,s,u\right)\right|\leq \varepsilon_{1}$ and $\left|H\left(t,s,u\right)-\tilde{H}\left(t,s,u\right)\right|\leq \varepsilon_{2}$, for all *u* ∈ *R* and for all *t*, *s* ∈ [*a*_1_; *b*_1_] × ⋯×[*a*_*m*_; *b*_*m*_].

If x* and ${\tilde{x}}^{*}$ are solutions of corresponding equations (15) and (16), respectively, then one has that

(18) $$\begin{array}{c} ||x^{*}-{\tilde{x}}^{*}||\leq \frac{3\left(\beta \varepsilon_{1}+\gamma \varepsilon_{2}\right)\prod_{i=1}^{m}\left(b_{i}-a_{i}\right)}{1-\left[\alpha +\left(\beta L_{K}+\gamma L_{H}\right)\prod_{i=1}^{m}\left(b_{i}-a_{i}\right)\right]}. \end{array}$$

**Proof**

Using (1), (15), (16), (17), and assumptions (*A*_1_)–(*A*_4_) and (iii), we obtain

(19) ||xn+1−x~n+1||=||Tyn−T~y~n||=|F(t,yn(t),∫a1t1⋯∫amtmK(t,s,yn(s))ds, ∫a1b1⋯∫ambmH(t,s,yn(s))ds) −F(t,y~n(t),∫a1t1⋯∫amtmK~(t,s,y~n(s))ds, ∫a1b1⋯∫ambmH~(t,s,y~n(s))ds)|≤α|yn(t)−y~n(t)| +β∫a1t1⋯∫amtm|K(t,s,yn(s))−K~(t,s,y~n(s))|ds +γ∫a1b1⋯∫ambm|H(t,s,yn(s))−H~(t,s,y~n(s))|ds≤α|yn(t)−y~n(t)| +β∫a1t1⋯∫amtm(|K(t,s,yn(s))−K(t,s,y~n(s))| +|K(t,s,y~n(s))−K~(t,s,y~n(s))|)ds +γ∫a1b1⋯∫ambm(|H(t,s,yn(s))−H(t,s,y~n(s))| +|H(t,s,y~n(s))−H~(t,s,y~n(s))|)ds≤α|yn(t)−y~n(t)| +β∫a1t1⋯∫amtm(LK|yn(s)−y~n(s)|+ε1)ds +γ∫a1b1⋯∫ambm(LH|yn(s)−y~n(s)|+ε2)ds≤α||yn−y~n||+β(LK||yn−y~n||+ε1)∏i=1m(bi−ai) +γ(LH||yn−y~n||+ε2)∏i=1m(bi−ai)≤[α+(βLK+γLH)∏i=1m(bi−ai)]||yn−y~n|| +(βε1+γε2)∏i=1m(bi−ai),

(20) ||yn−y~n||≤(1−ξn)|xn(t)−x~n(t)| +ξn|T(xn)(t)−T~(x~n)(t)|≤(1−ξn)|xn(t)−x~n(t)| +ξn{α|xn(t)−x~n(t)| +β∫a1t1⋯∫amtm{LK|xn(s)−x~n(s)|+ε1}ds +γ∫a1b1⋯∫ambm{LH|xn(s)−x~n(s)|+ε2}ds}≤{1−ξn(1−[α+(βLK+γLH)∏i=1m(bi−ai)])} ×||xn−x~n||+ξn(βε1+γε2)∏i=1m(bi−ai).

Combining (19) with (20) and using assumptions (*A*_5_) and 1/2 ≤ *ξ*_*n*_ in the resulting inequality, we get

(21) ||xn+1−x~n+1||≤{1−ξn(1−[α+(βLK+γLH)∏i=1m(bi−ai)])} ×||xn−x~n|| +ξn(1−[α+(βLK+γLH)∏i=1m(bi−ai)]) ×3(βε1+γε2)∏i=1m(bi−ai)1−[α+(βLK+γLH)∏i=1m(bi−ai)].

Denote that

(22) βn=||xn−x~n||,μn=ξn(1−[α+(βLK+γLH)∏i=1m(bi−ai)])∈(0,1),γn=3(βε1+γε2)∏i=1m(bi−ai)1−[α+(βLK+γLH)∏i=1m(bi−ai)]≥0.

It is clear that inequality (21) satisfies all conditions in Lemma 3, and hence it follows that

(23) $$\begin{array}{c} ||x^{*}-{\tilde{x}}^{*}||\leq \frac{3\left(\beta \varepsilon_{1}+\gamma \varepsilon_{2}\right)\prod_{i=1}^{m}\left(b_{i}-a_{i}\right)}{1-\left[\alpha +\left(\beta L_{K}+\gamma L_{H}\right)\prod_{i=1}^{m}\left(b_{i}-a_{i}\right)\right]}. \end{array}$$
